# Supplementary material for: Cellular macromolecules-tethered DNA walking indexing to explore nanoenvironments of chromatin modifications
Source: Nat Commun. 2021 Mar 30;12:1965. doi: 10.1038/s41467-021-22284-z (PMC8009891; doi:10.1038/s41467-021-22284-z)
Supplement: Supplementary file 1 — Supplementary Information [file 41467_2021_22284_MOESM1_ESM.pdf]

Supplementary Information

Cellular Macromolecules-Tethered DNA Walking Indexing to Explore Nanoenvironments of Chromatin Modifications

Feng Chen[1, ‡], Min Bai[1, ‡], Xiaowen Cao[1], Jing Xue[1], Yue Zhao[1], Na Wu[1], Lei Wang[2], Dexin Zhang[3] and Yongxi Zhao[1,*]

[1]Institute of Analytical Chemistry and Instrument for Life Science, The Key Laboratory of Biomedical Information Engineering of Ministry of Education, School of Life Science and Technology, Xi'an Jiaotong University, Xianning West Road, Xi'an, Shaanxi, P. R. China

[2]Department of Thoracic Surgery, Tangdu Hospital, Air Force Medical University, Xi'an, Shaanxi, P. R. China

[3]Department of Respiratory Medicine, The Second Affiliated Hospital of Medical College, Xi'an Jiaotong University, Xiwu Road, Xi'an, Shaanxi, P. R. China

[‡]These authors contributed equally to this work. Correspondence and requests for materials should be addressed to Y.X.Z. (yxzhao@mail.xjtu.edu.cn)

Table of Contents

Supplementary Table 1. Sequence information for oligonucleotides used in Cell-TALKING **P3**

Supplementary Figure 1. Other images of the experiments of in vitro Cell-TALKING performed on coverglass **P4**

Supplementary Figure 2. Performance of in vitro Cell-TALKING on DNA origami substrates **P5**

Supplementary Figure 3. The structural characterization of ATP-γ-alkyne **P6**

Supplementary Figure 4. Efficient labelling and imaging of DNA modifications in cells **P7**

Supplementary Figure 5. Specific cell imaging using Cell-TALKING **P8**

Supplementary Figure 6. Statistical analysis of fluorescence intensity and spot count in single cells corresponding to Figure 3A **P9**

Supplementary Figure 7. Comparison of PLA and Cell-TALKING in the detection of single combination modification **P10**

Supplementary Figure 8. 3D overlap analysis of the spots from different fluorescence channels in the image used in Figure 3C **P11**

Supplementary Figure 9. Statistical analysis of nanoenvironments of chromatin modifications during cell cycles **P12**

Supplementary Figure 10. Statistical analysis of spot count and fluorescence intensity in single cells corresponding to Figure 5A **P13**

Supplementary materials and the protocol of Cell-TALKING to explore nanoenvironments of chromatin modifications P14

Supplementary Table 1. Sequence information for oligonucleotides used in Cell-TALKING**.**

Name	Sequences (5′-3′)
	**Oligonucleotides for lableing chromatin modifications in cells**
Walking probe	AGTCAGAGTCAGAGTGTAGACAGTAAAAAAAAAAAAAAAAAAAAAACT***GCTGAGG***CACAT
Walking blocker	TATGTGCCTCAGCAGAGT
Non-walking probe	AGTCAGAGTCAGAGTGTAGACAGTAAAAAAAAAAAAAAAAAAAAAACTCAAAGAACACAT
Antibody-crosslinked probe	**N_3**-ACTGTCTACACTCTGACTCTGACTAAAAAAAAAAAAA
Barcoding probe-5hmC	**DBCO-**ATCCTCGTAAATCCTCATCAATCA*T*CTATGTG***CCTCAGC***AGAGGAGA*A*T*C*A*C*/LNA_G/
Barcoding probe-5hmU	**N_3-**GGAGTGCAGCAAACGGGAAGAGTC*T*TTATGTG***CCTCAGC***AGAGGAGA*A*T*C*A*C*/LNA_G/
Barcoding probe -5fU	**N_3-**GCTATGTTTCTTGAGGAGGGCAGC*A*ATATGTG***CCTCAGC***AGAGGAGA*A*T*C*A*C*/LNA_G/
	**Oligonucleotides for RCA and fluorescence imaging**
Padlock probe-5hmC	**P-**GAGGATTTACTCGACAGAGCTTACTCACAGCCAGCATCACAAGGTCGATGATTGAT
Ligation linker-5hmC	GTAAATCCTCATCAATCATC
Padlock probe-5hmU	**P-**CCGTTTGCTGTGGCCTGAGCCTTCCTCGGTACGGTCTGTAAGGTCAAGACTCTTC
Ligation linker-5hmU	CAGCAAACGGGAAGAGTCTT
Padlock probe-5fU	**P-**CCTCAAGAAATCGACGCGTATAATAATGCTGGGCTCTAGTAGGTCTTGCTGCCCT
Ligation linker -5fU	TTTCTTGAGGAGGGCAGCAA
Cy3-probe	**Cy3**-AGAGCTTACTCACAGCCAGCATCACA
Cy5-probe	**Cy5**-TGAGCCTTCCTCGGTACGGTCTGTA
FAM-probe	GCGTATAATAATGCTGGGCTCTAGT-**FAM**
Mismatched padlock probe	**P-**GCGGTAGTCTGATGCTGAGCCTTCCTCGGTACGGTCTGTATCAGAGAACAATTTG
Ligation linker for mismatched padlock probe	AGACTACCGCCAAATTGTTC
Complementary probe for Barcoding probe-5hmC	GGCACATAGATGATTGATGAGGATTTACGAGGAT
Complementary probe for Barcoding probe-5hmU	GGCACATAAAGACTCTTCCCGTTTGCTGCACTCC
Complementary probe for Barcoding probe-5fU	GGCACATATTGCTGCCCTCCTCAAGAAACATAGC
	**Oligonucleotides for preparing dsDNA substrates containing modified U**
Primer-modified U	GCGATCAGCCTGTGCAGTTGTCGTACGACCA
Template-modified U	CT**G**CCATGGTCGTACGATTTTTT
	**Oligonucleotides for the experiments on coverglass**
Glass-Capture substrate	ACTCGGAAGGAGCCATGCCAGACATTCCTGTCCGAAGTCCCGGGGAACGTAACTCATGTCAAGGTACACTCTGACTCTGACTCAACGATTATAACAGTTTTT
Glass-Walking probe	AGTCAGAGTCAGAGTGTAGACAGTAAAAAAAAAAAAAAAAAAAAAAAAAAAAAAAAAAAAAAAAAAAAACT***GCTGAGG***CACAT
Glass-Barcoding probe-5hmC	AAACTGTTATAATCGTTGTCCTCGTAAATCCTCATCAATCA*T*CTATGTG***CCTCAGC***AGAGGAGA*A*T*C*A*C*G
Glass-Barcoding probe-5hmU	CCTTGACATGAGTTACGTGAGTGCAGCAAACGGGAAGAGTC*T*TTATGTG***CCTCAGC***AGAGGAGA*A*T*C*A*C*G
Glass-Barcoding probe-5fU	TCCCCGGGACTTCGGACACTATGTTTCTTGAGGAGGGCAGC*A*ATATGTG***CCTCAGC***AGAGGAGA*A*T*C*A*C*G
Glass-Modified primer	GGAATGTCTGGCATGGCTCCTTCCGAGT-**Maleimide**
	**RCA primers for testing bioorthogonal labelling of 5hmU and 5fU in cells**
5hmU-primer probe	**N_3-**GGAGTGCAGCAAACGGGAAGAGTCTT
5fU-primer probe	**N_3-**GCTATGTTTCTTGAGGAGGGCAGCAA

The letter '**P**' and the symbol '*' indicate phosphate group and phosphorothioate, respectively. The /LNA_G/ indicate the G base of locked nucleic acid (LNA). Italic letters in red indicate the nick sequence of Nt.BbvCI enzyme.

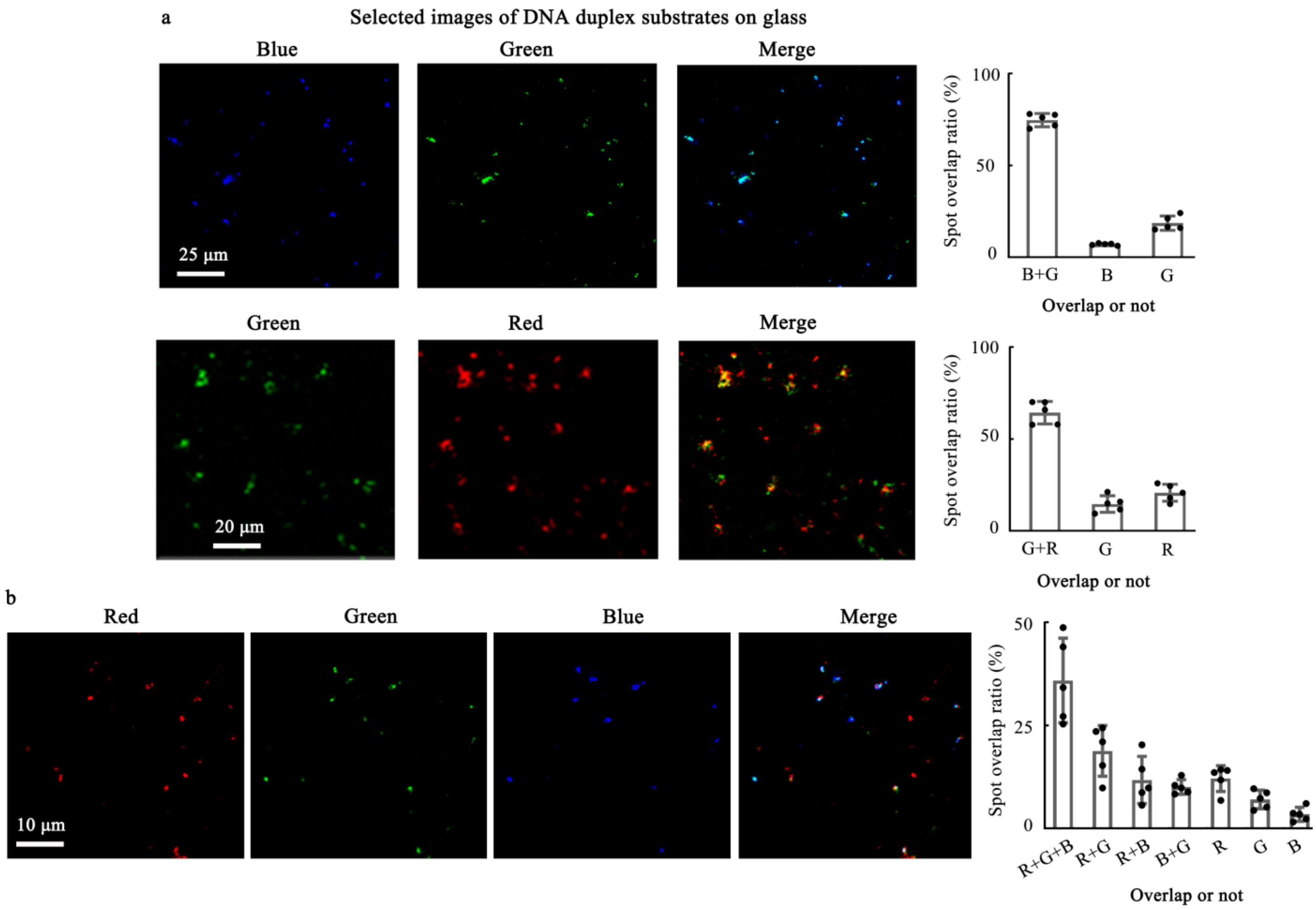

Supplementary Figure 1. Other images of the experiments of *in vitro* Cell-TALKING performed on coverglass. (a) and (b) are corresponding to Figure 2a and 2b, respectively. Five repeated experiments were performed for statistical analysis of spot count of randomly selected images. The "spot overlap ratio" means the proportion of the spot count of one kind (fluorescence overlaps or not) in total number of all kinds. The letters B, G, and R indicate spots of blue, green, and red, respectively. There are some spots that fail to overlap two or three fluorescence signals, indicating the false negative response of our method on this DNA substrate on glass. It is disccussed in the main text. The data of bar charts are presented as mean values +/- SD.

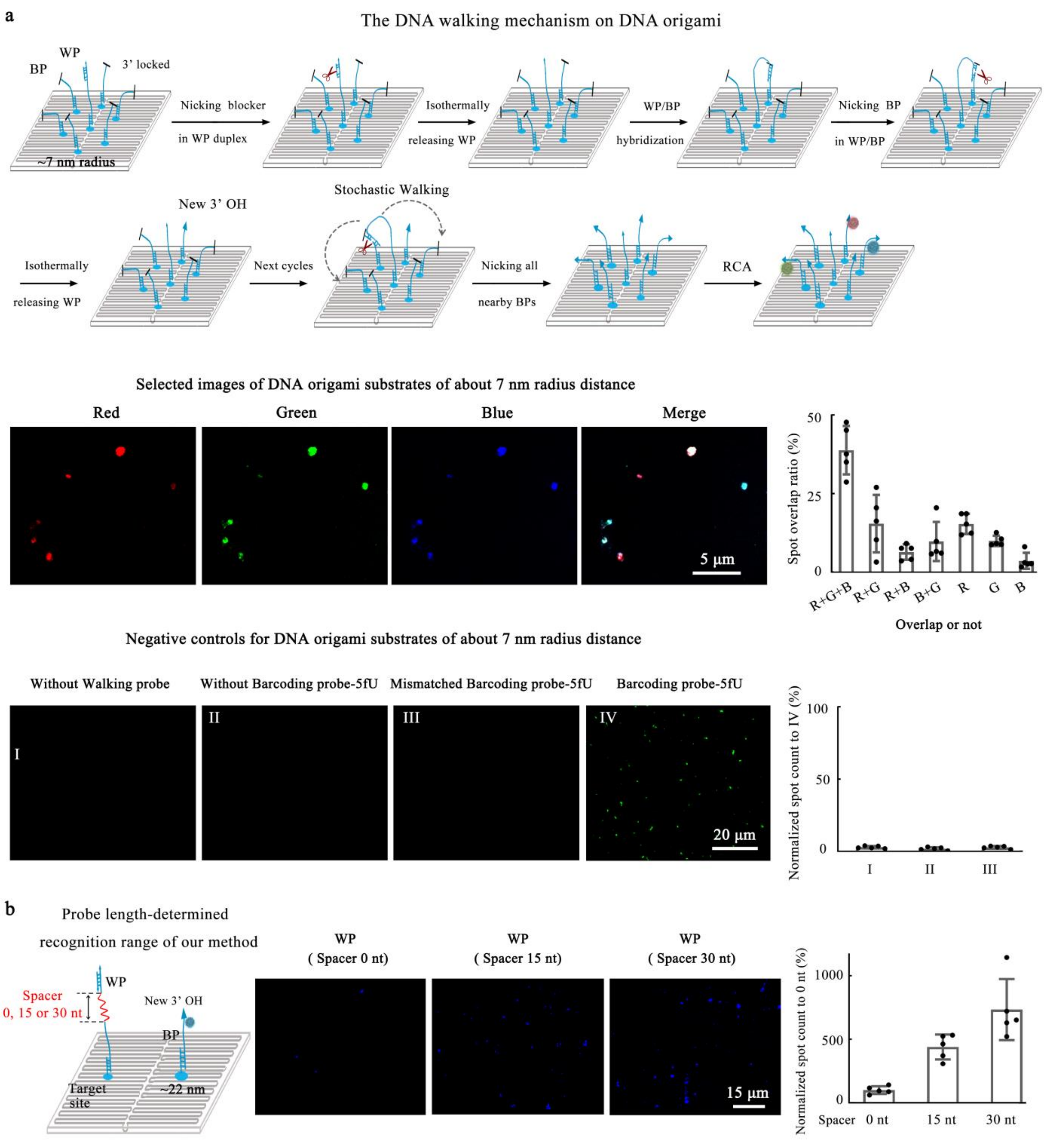

Supplementary Figure 2. Performance of *in vitro* Cell-TALKING on DNA origami substrates. (a) Upper, the actual walking mechanism. Two copies of three barcoding probes (BPs) surround the blocked walking probe (WP) with about 7 nm radius distance on one origami molecule. During the nicking/walking process, the nicking enzyme firstly cuts the blocker sequence in the blocked WP duplex. Then the walking probe molecule in this duplex can be isothermally released to hybridize one BP molecule, forming a DNA duplex as the substrate for nicking enzyme. After cuting this BP molecule in the duplex, the walking probe molecule will walk to and hybridize another BP molecule. In this way, all these BP molecules can be cleaved and the newly generated 3'-OH ends can induce RCA reaction .Middle, statistical analysis of false negative response of our method. The images are corresponding to Figure 2c. Bottom, investigating the false positive response by several negative controls. There are some spots that fail to overlap two or three fluorescence signals, indicating the false negative response of our method on this DNA origami substrate. It is disccussed in the main text. (b) Investigating the probe length-determined recognition range of our method using different walking probes and DNA origami substrates. Five repeated experiments were performed for statistical analysis of spot count of randomly selected images. The "normalized spot count" means the spot count of one sample relative to that of the given one (e.g., the positive control (IV) in Supplementary Figure 2a or the sample using walking probe containing 0 nt spacer in Supplementary Figure 2b). The data of bar charts are presented as mean values +/- SD.

^1H NMR

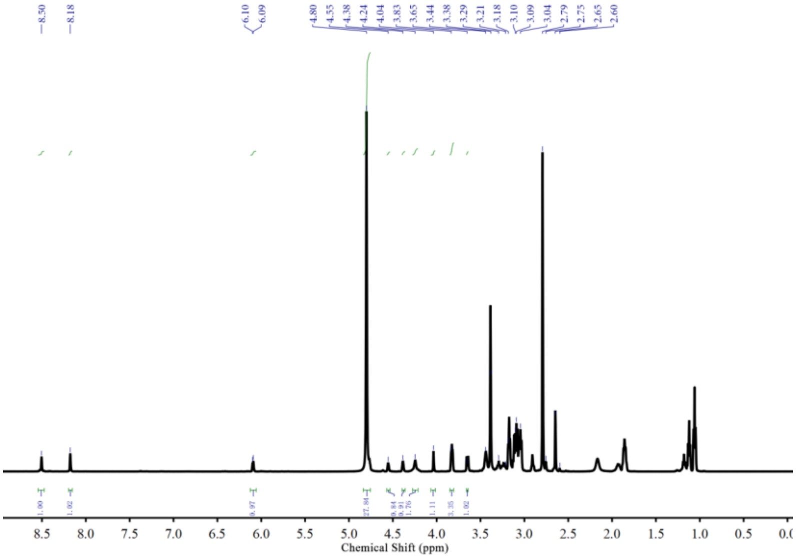

^{13}C NMR

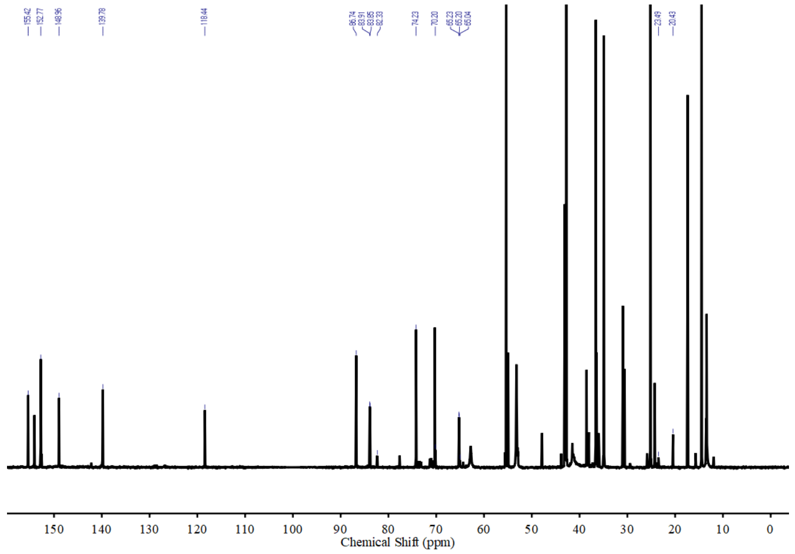

^{31}P NMR

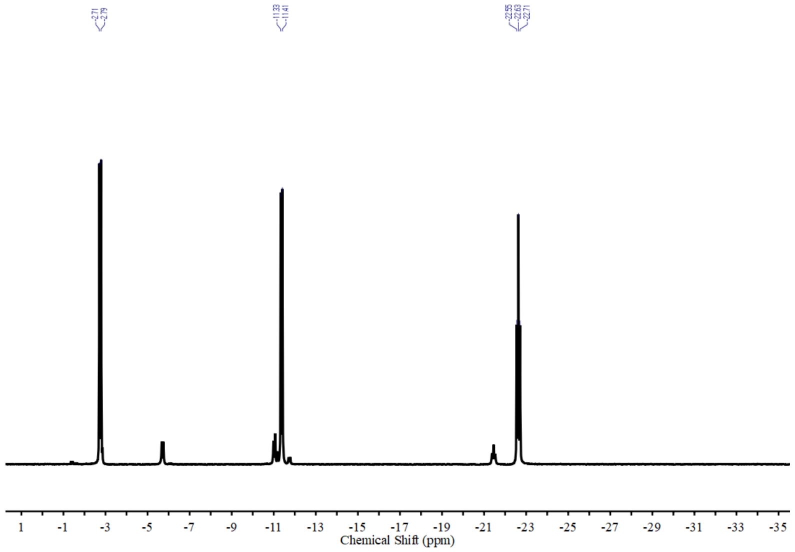

Supplementary Figure 3. The structural characterization of ATP-γ-alkyne. See more details in the following Supplementary Materials.

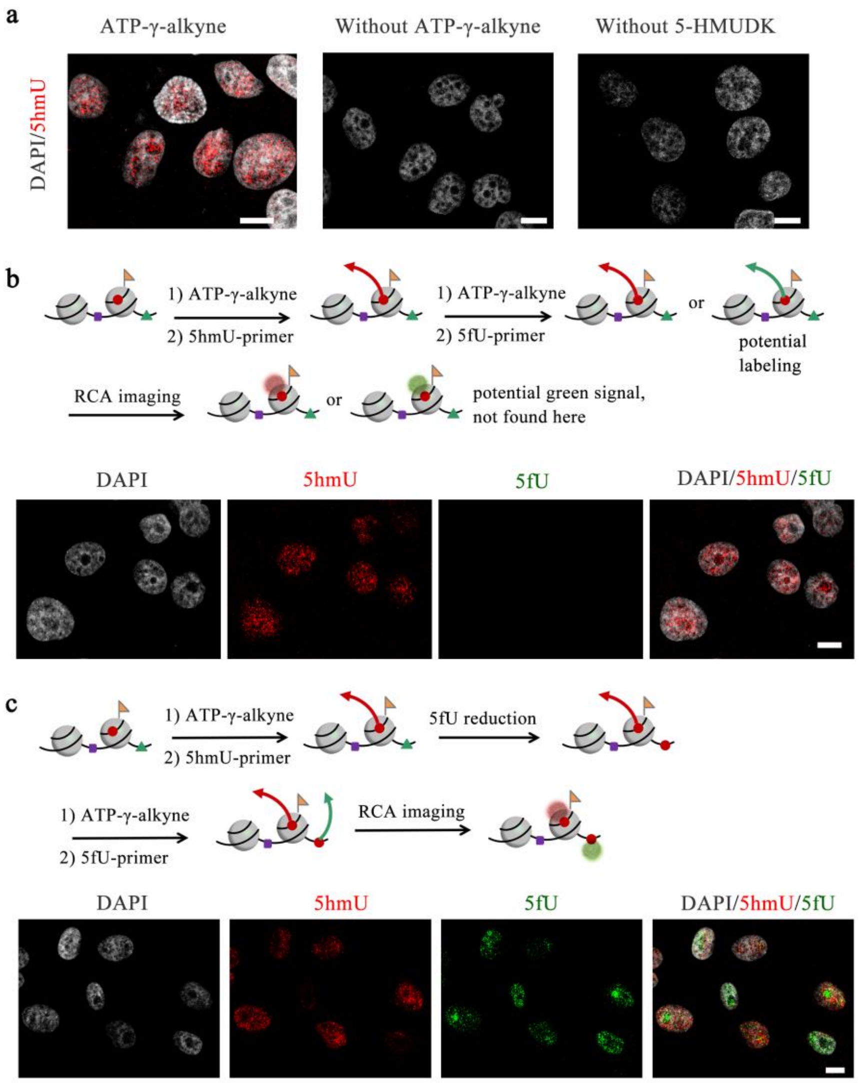

Supplementary Figure 4. Efficient labelling and imaging of DNA modifications in cells. (a) Bioorthogonal labelling of 5hmU in cells using 5-HMUDK and ATP-γ-alkyne. (b) Almost complete crosslinking of 5hmU sites in cells with the 5hmU-primer probe. After the click reaction of the 5hmU-primer probe, the 5fU-primer probe was subsequently added. No RCA fluorescence signal of 5fU-primer probe was observed. It indicated all sites of 5hmU modification were crosslinked with the 5hmU-primer probe. (c) Successive labelling of 5hmU and 5fU in cells and simultaneous imaging analysis. MCF-10A cells were used. Scale bar, 10 µm. For the experiments of cell imaging in all figures of this work, five times were repeated independently with similar results.

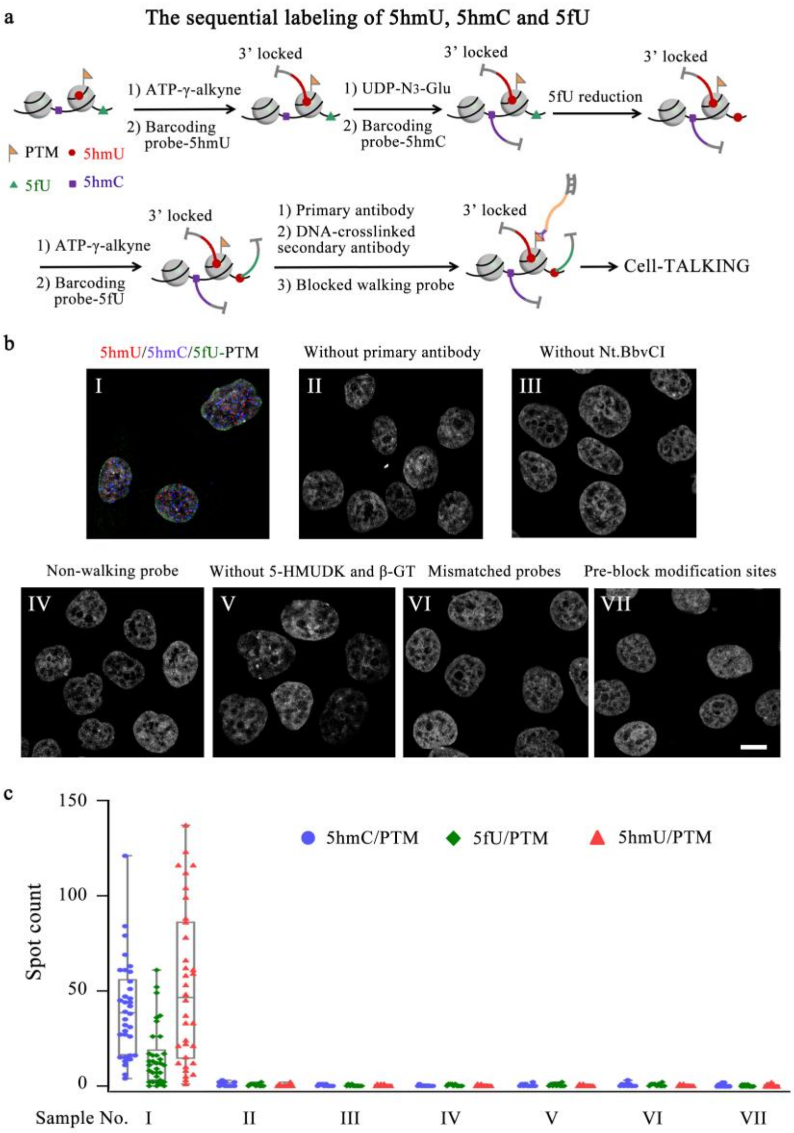

Supplementary Figure 5. Specific cell imaging using Cell-TALKING. (a) Scheme of sequential labeling of 5hmU, 5hmC and 5fU followed by the histone PTM in cells. The blocked walking probe is a DNA hybrid duplex that can be captured by the DNA-crosslinked secondary antibody. The detail is described in the protocol of Cell-TALKING as below. (b) Selected cell images for several negative controls and one positive sample. The nanoenvironment around histone H3K27ac in MCF-10A cells was detected here. Scale bar, 10 μm. (c) The spot counts of three combination sites of single cells. The cell numbers of Sample I-VII are 34, 10, 10, 10, 10, 10 and 10, respectively. The "Non-walking probe" negative control used the Non-walking probe instead of the walking probe. The Non-walking probe can not hybridize to all three barcoding probes, which fails to induce the DNA nicking reaction and walking process. The "Mismatched probes" negative control used a circularized padlock that can not hybridize to all three barcoding probes, which fails to induce RCA. And the "Pre-block modification sites" control firstly labeled all modification sites with blocked duplex barcoding probes, and then with single-stranded barcoding probes. The blocked duplex barcoding probes were prepared by the hybridization of the complementary oligo to the single-stranded barcoding probes. They can not hybridize to their circularized padlocks and fail to induce RCA. This negative control can indicate that almost all sites were labeled and crosslinked with DNA probes by the first labeling processes, and no residual sites were detected by second labeling processes. Similar results are shown in Supplementary Figure 4. The maxima, upper quartile, median, lower quartile, and minima are shown in the box plots.

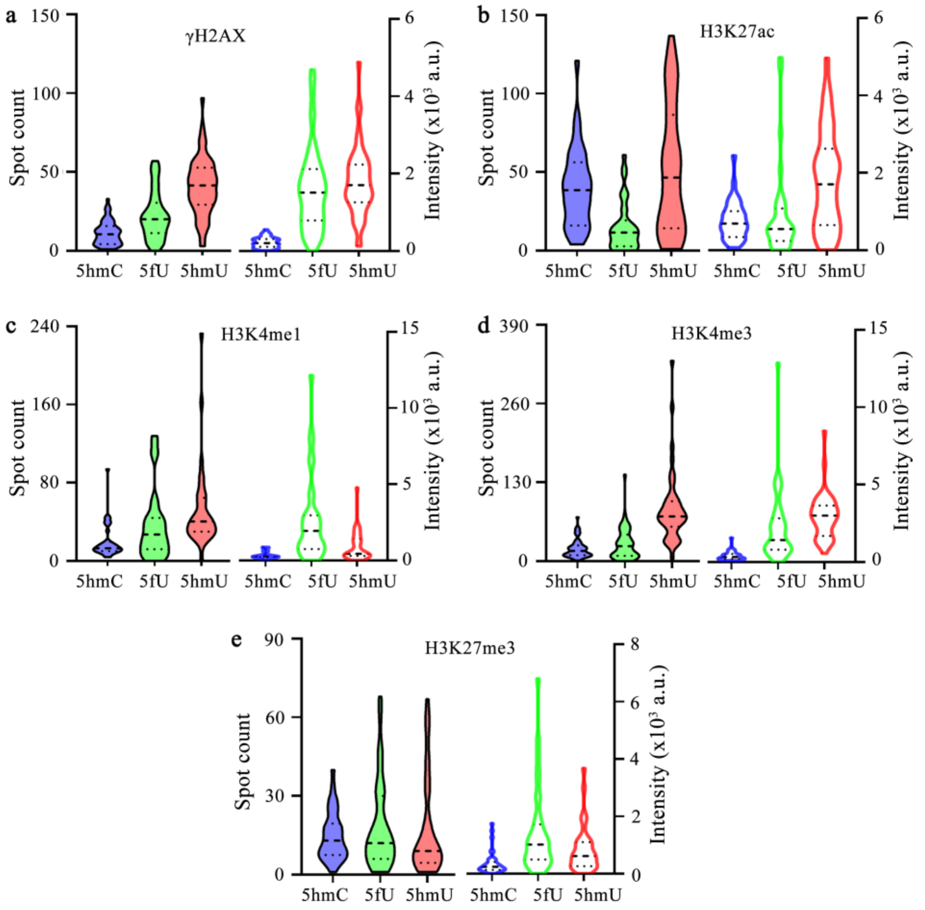

Supplementary Figure 6. Statistical analysis of fluorescence intensity and spot count in single cells of each channel corresponding to Figure 4a. MCF-10A was used. The upper quartile, median, and lower quartile are shown in all violin plots in this work.

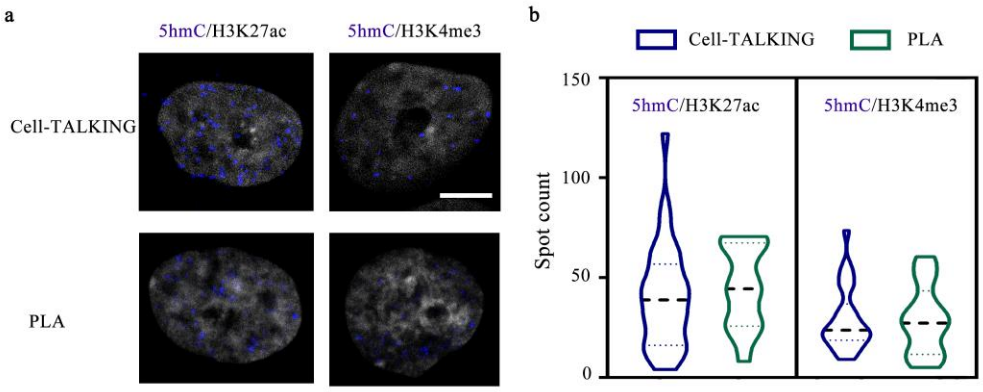

Supplementary Figure 7. Comparison of PLA and Cell-TALKING in the detection of single combination modification. (a) Selected merged cell images. The scale bar is 10 μm. (b) The spot counts of single cells from these samples (cell numbers are about 50). It can be observed that similar detection results were provided by these two methods in both testing targets. It confirmed that the efficacy of our method was well consistent with that of PLA.

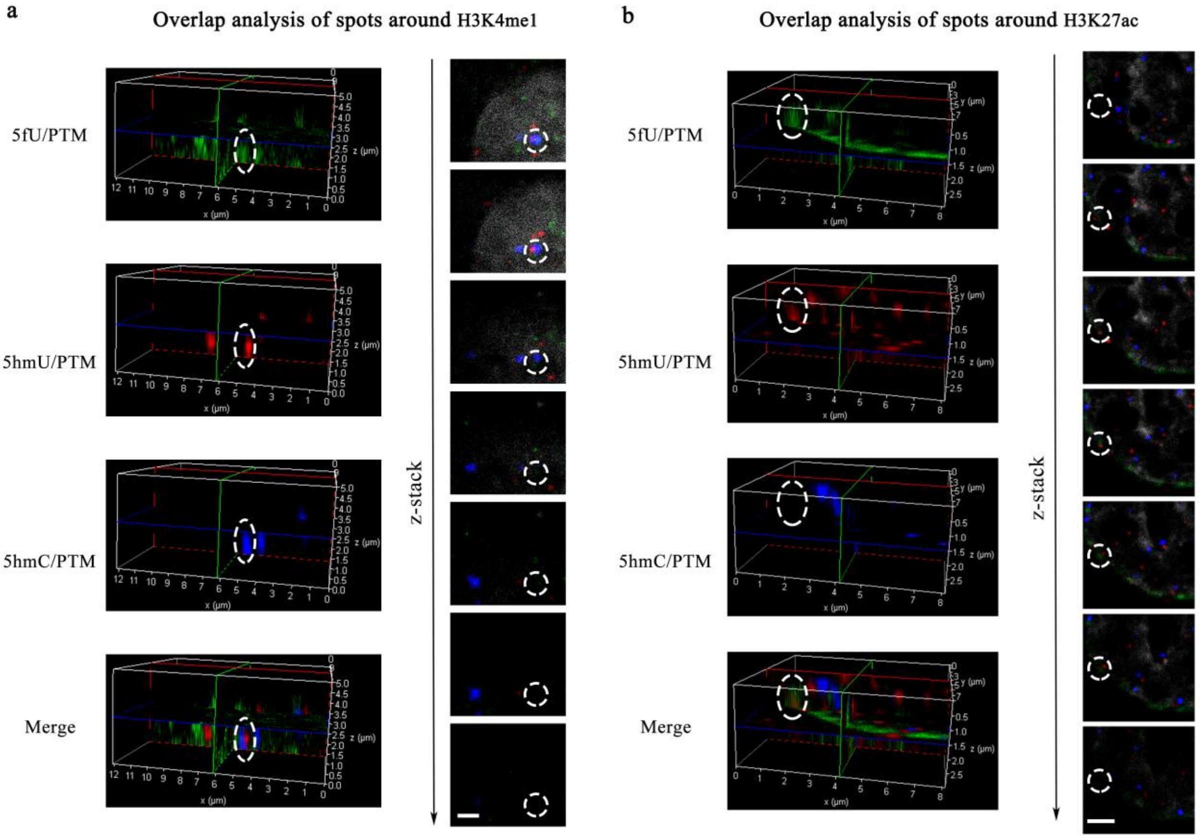

Supplementary Figure 8. 3D overlap analysis of the spots from different fluorescence channels in the image used in Figure 4c. The images were acquired in 3D with the z-stack at intervals of 0.5 μm. The spots highlighted in circle are overlapped in three or two fluorescence channels with the spatial resolution of hundreds of nanometers. The scale bars are 1 μm.

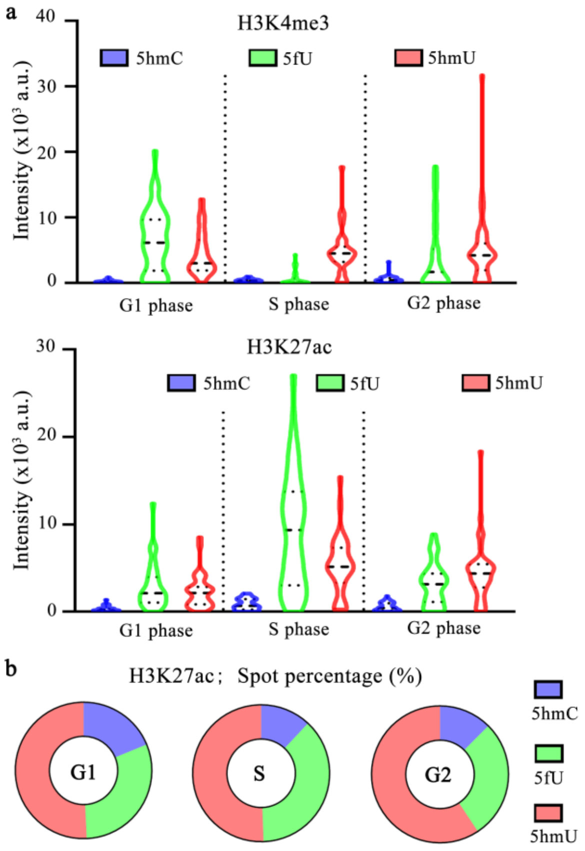

Supplementary Figure 9. Statistical analysis of nanoenvironments of chromatin modifications during cell cycles. (a) Statistical analysis of fluorescence intensity of three combination patterns in single cells of each channel corresponding to Figure 5a. (b) The spot percentages of three combination patterns around H3K27ac during cell cycles. MCF-10A was used.

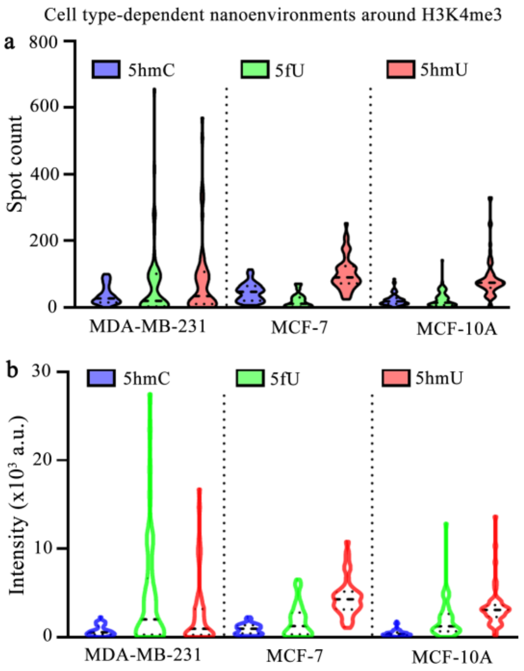

Supplementary Figure 10. Statistical analysis of spot count and fluorescence intensity in single cells of each channel corresponding to Figure 6a. MCF-10A was used here.

Supplementary materials and the protocol of Cell-TALKING to explore nanoenvironments of chromatin modifications

Chemical synthesis and characterization of ATP-γ-alkyne. Adenosine 5´-triphosphate (ATP) disodium salt (Sigma Aldrich, cat. no. 10519979001) and propargylamine (Sigma Aldrich, cat. no. P50900) were used to synthesize ATP-γ-alkyne. ATP disodium salt (4.9 mM) and propargylamine (245 mM) were dissolved in water. And then the pH was adjusted to 6.0. 490 mM EDC-HCl (Sigma Aldrich, cat. no. E6383) was added to the above solution, and the mixture was stirred for 24 h at room temperature. Then the pH was adjusted to 8.5. After evaporating, the light yellow viscous products was purified with silica gel column chromatography to get the white solid ATP-γ-alkyne. ATP: HRMS (ESI, negative mode) for $C_{10}H_{15}N_5O_{13}P_3$, $[M+H]^-$: 505.98847 (calculated), 505.98488 (found). ATP-γ-alkyne: HRMS (ESI, negative mode) for $C_{13}H_{18}N_6O_{12}P_3$, $[M+H]^-$: 543.02010 (calculated), 543.01591 (found). All the mass spectrometry analyses in this work were performed on a Waters I-Class Vion IMS Qtof with electrospray ionization.

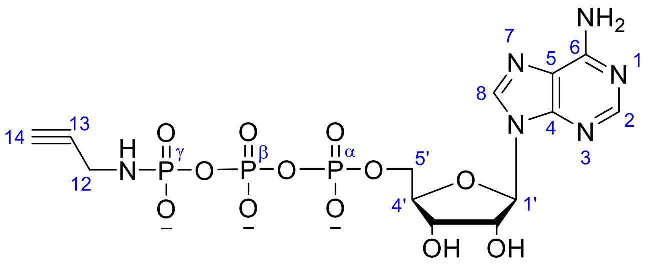

NMR Spectra were recorded on a Brucker AVANCE III HD 600 MHz machine. The deuterated solvent in this work was D_2O. ^{31}P NMR spectra are referenced to H_3PO_4 as an external standard. ^{13}C NMR spectra are referenced to tetramethylsilane (TMS) as an external standard. Chemical shifts (δ) are quoted in parts per million (ppm) and coupling constants (J) are measured in hertz (Hz). The following abbreviations are used to describe multiplicities: s=singlet, d=doublet, t=triplet, m = multiplet. The spectra are shown in Figure S3.

^{1}H NMR (600 MHz, D_2O): δ (ppm) = 8.50 (s, 1H, 2-H), 8.18 (s, 1H, H8), 6.10 (d, *J*=6.09 Hz, 1H, H1´), 4.80 (m, 1H,overlapping with HOD, H2´) ,4.62-4.55 (m, 1H, H3´), 4.38 (m, 1H, H4´), 4.24 (m, 2H, H5´), 2.79-2.65 (m, 3H, H12, H14).

^{13}C NMR (150 MHz, D_2O): *δ* (ppm) = 155.4 (C6), 152.7 (C2), 149.0 (C4), 139.8 (C8), 118.4 (C5), 86.7 (C1´), 83.9 ((d, J_{PC}=9.0 Hz, C4´), 82.3 (C13), 74.2 (C2´), 70.3 (C3´), 70.2 (C14), 65.2 (d, J_{PC} =5.6 Hz, C5´), 23.5 (C12).

^{31}P NMR (243 MHz, D_2O): *δ* (ppm) = -2.75 (d, *J* =20.5 Hz, γ), -11.37 (d, *J* = 19.1 Hz, α), -22.63 (t, *J* = 19.6 Hz, β).

IR measurements were performed on Bruker VERTEX70 Micro-Infrared Spectroscopy equipped with a diamond-ATR setup. IR (ATR): ṽ (cm-1) = 3249, 2976, 2680, 2472, 2119, 1620, 1562, 1481, 1384, 1338, 1261, 1164, 1082, 1047, 881, 599.

PDMS chamber preparation. The chip was fabricated via pouring poly(dimethylsiloxane) (PDMS, oligomer and cross-linker at a ratio of 10:1 w/w, Dow Corning, Germany) onto the silicon. After degassed and cured at 70 °C for 3 h, the slab of cured PDMS containing independent wells (φ 4 mm) was cut and the flat side was adhered to

the coverslips using plasma cleaner (Harrick plasma, PDC-002-HP), forming the chambers for cell culture and following chemical reactions.

Preparation of DNA-crosslinked secondary antibody. 50 μg donkey anti-Rabbit IgG (H+L) highly cross-adsorbed secondary antibody (ThermoFisher Scientific, cat. no. A16037) was conjugated with 0.5 mM Dibenzocyclooctyne-sulfo-N-hydroxysuccinimidyl ester (DBCO-sulfo-NHS ester) (Sigma Aldrich, cat. no. 762040) in 1x PBS at room temperature for 1 h with rotation. The product was purified by Amicon Ultra-0.5 NMWL 30 KDa centrifugal filter (Merck Millipore). Then 20 μM antibody-crosslinked probe was mixed with purified DBCO-conjugated antibody, and the incubation reaction was performed at 4 ^{o}C for 1 week with gentle rotation. The sample was purified using an Amicon Ultra-0.5 NMWL 100 KDa centrifugal filter (Merck Millipore). The resulted antibody/DNA conjugateds were stored at 4 ^{o}C.

Cell culture. MCF-10A, MCF-7 and MDA-MB-231 cells were cultured in Dulbecco's modified Eagle's medium with 10% fetal-bovine serum and 1% antibiotics penicillin-streptomycin (100 U/mL) in a humidified incubator containing CO_2 (5%) at 37 ^{o}C. For a typical experiment, 6,000 cells were seeded on a collagen A-coated coverslip enclosed in a PDMS chamber at 37 ^{o}C overnight.

Cell fixation and permeabilization. The cells cultured on the PDMS chamber were fixed with 4% formaldehyde (Beyotime Biotechnology, cat. no. P0099) at room temperature for 10 min, and then were permeabilized with 0.5% Triton X-100 (Sangon Biological, cat. no. A110694) in 1x PBS for 5 min at room temperature.

The labeling of DNA modifications. We follow the labelling order (5hmU first, then 5hmC and finally 5fU) to achieve the discrimination of these three DNA modifications in cells.

(1-1). 5hmU phosphorylation reaction

The cells were incubated with following mixture at 37 ^{o}C for 2 h then washed with 1x PBS buffer for three times.

Reagents	μL
5-HMUDK (New England Biolabs Ltd., cat. no. M0659S)	1
ATP-γ-alkyne (10 mM)	1
10x Cutsmart buffer	2
Water	16
Total	20

(1-2). The labeling with barcoding probe-5hmU

The following solution was added to perform a copper(I)-catalyzed click reaction at room temperature for 1 h in dark.

Reagents	μL
Barcoding probe-5hmU (2 mM)	2.5
Copper(II) sulfate (20 mM, Aladdin, cat. no. C119000)	1

Sperm DNA (10 μg/mL)	1
Sodium ascorbate (500 mM, Aladdin, cat. no. S105026)	4
20x PBS	1
Water	10.5
Total	20

(2-1). 5hmC glycosylation reaction

After careful washing of cell samples, the glycosylation reaction of 5hmC was performed. The following mixture with cells was incubated at 37 °C for 2 h. Then the cells were washed with 1x PBS buffer for three times.

Reagents	μL
T4 β-GT (New England Biolabs Ltd., cat. no. M0357S)	1
UDP-N_3-Glu (1 mM, Jena Bioscience, cat. no. CLK-076)	1
10x NEBuffer 4	2
Water	16
Total	20

(2-2). The labeling with barcoding probe-5hmC

The following mixture was added to perform a copper-free click reaction at 37 °C for 60 min. Then the cells were washed with 1x PBS buffer for three times.

Reagents	μL
Barcoding probe-5hmC (2 mM)	1
Sperm DNA (10 μg/mL)	1
20x PBS	1
Water	17
Total	20

(3-1). The reduction of 5fU to 5hmU

The cells were incubated with following mixture at room temperature in dark. Then the cells were washed with 1x PBS buffer for three times.

Reagents	μL
Sodium borohydride (10 mg/mL, Aladdin, cat. no. S108355)	2
Water	18
Total	20

(3-2). The phosphorylation of newly generated 5hmU

The cells were incubated with following mixture at 37 °C for 2 h then washed with 1x PBS buffer for three times.

Reagents	μL
5-HMUDK (New England Biolabs Ltd., cat. no. M0659S)	1
ATP-γ-alkyne (10 mM)	1
10x Cutsmart buffer	2
Water	16
Total	20

(3-3). The labeling with barcoding probe-5fU

The following solution was added to perform a copper(I)-catalyzed click reaction at room temperature for 1 h in dark. Then the cells were washed with 1x PBS buffer for three times.

Reagents	μL
Barcoding probe-5fU (2 mM)	2.5
Copper(II) sulfate (20 mM, Aladdin, cat. no. C119000)	1
Sperm DNA (10 μg/mL)	1
Sodium ascorbate (500 mM, Aladdin, cat. no. S105026)	4
20x PBS	1
Water	10.5
Total	20

The labeling of histone PTM with primary antibody. First, the cells in chamber were blocked with QuickBlock™ blocking buffer (Beyotime Biotechnology, cat. no. P0260) at room temperature for 1 h. Then the cells were incubated with different primary antibody (Cell Signaling Technology), H3K4me1 (1:800), H3K4me3 (1:400), H3K27me3 (1:1600), γH2AX (1:400) and H3K27ac (1:100) diluted by QuickBlock™ Primary Antibody Dilution Buffer (Beyotime Biotechnology, cat. no. P0262) for overnight at 4 ºC. Wash the cells with 1x PBST buffer (0.01% Tween-20 in PBS) three times for at least 10 minutes each time.

The labeling with DNA-crosslinked secondary antibody. The cells were incubated with prepared DNA-crosslinked secondary antibody in QuickBlock™ Secondary antibody dilution buffer (Beyotime Biotechnology, cat. no. P0265) for 1h at room temperature. The cells were washed using PBST buffer three times for at least 15 minutes each time. Notably, this DNA sequence crosslinked on secondary antibody is used to capture the blocked walking probe via DNA hybridization. This design can regulate the length of walking probe without the change of DNA-crosslinked secondary antibody. And the blocked walking probe is a DNA hybrid duplex prepared as below.

The preparation of the blocked walking probe duplex

The following mixture was incubated in PCR tube at 37 ºC for 2 h.

Reagents	μL

Walking probe (20 μM)	5
Walking blocker (20 μM)	5
10x NEBuffer 2	5
Water	35
Total	50

The hybridization of the blocked walking probe duplex

The cell samples were incubated with following mixture at 37 ^{o}C for 1 h then washed with 2x SSC buffer for three times.

Reagents	μL
Blocked walking probe (2 μM)	1
Sperm DNA (10 μg/mL)	1
Formamide buffer	4
20x SSC	2
Water	12
Total	20

Enzymatic and DNA reactions of Cell-TALKING. After the above labelling reactions, the Cell-TALKING was performed, which included the DNA proximity nicking/walking reaction, the hybridization reaction of circularized padlocks, 3' to 5' digestion and RCA by Phi 29 DNA polymerase, and the hybridization of fluorophore-labeled DNA probes.

(1). DNA proximity nicking/walking reaction

The cells were incubated with following mixture at 37 ^{o}C for 2 h then washed with 1x PBST buffer for three times.

Reagents	μL
Nt.BbvCI nicking enzyme	
(New England Biolabs Ltd., cat. no. R0632S)	0.5
10x Cutsmart buffer	2
Water	17.5
Total	20

During this reaction, each walking probe molecule was isothermally released from the duplex of barcoding probe/walking probe, and was reused to recognize any nearby barcoding probes, and the cleaved barcoding probes formed new 3'-OH ends.

(2). Hybridization reaction of circularized padlocks

Then three circularized padlocks were added and captured by the barcoding probes. The following mixture was incubated at 37 ^{o}C for 3h. The cells were washed with 2x SSC buffer for three times.

Reagents	μL
Padlock probe-5hmU (2 μM)	2

Padlock probe-5hmC (2 μM)	2
Padlock probe-5fU (2 μM)	2
Formamide buffer	4
20x SSC	2
Water	8
Total	20

The ssDNA 3’-OH overhangs in the duplexes of circularized padlocks/cleaved barcoding probes can be degraded by phi 29 DNA polymerase with its inherent 3’ to 5’ proofreading exonuclease activity.

(3). Barcoding RCA

The cells were incubated with following mixture at 37 ^{o}C for 2 h.

Reagents	μL
Phi 29 DNA polymerase<br>(New England Biolabs Ltd., cat. no. M0269S)	0.5
10x phi29 DNA polymerase buffer	2
BSA (10 mg/mL)	0.5
dNTPs (10 mM)	2.5
Water	4.5
Total	10

(4) Hybridization of fluorophore-labeled DNA probes

After washing with 1x PBST, 200 nM of each fluorophore-labeled DNA probes in 2x SSC and 20% formamide buffer were incubated with the samples at 37 ^{o}C for 30 min. The nuclei were stained using DAPI. Cells were washed three times before fluorescence imaging.
