# Supplementary material for: Cellular macromolecules-tethered DNA walking indexing to explore nanoenvironments of chromatin modifications
Source: Nat Commun. 2021 Mar 30;12:1965. doi: 10.1038/s41467-021-22284-z (PMC8009891; doi:10.1038/s41467-021-22284-z)
Supplement: Supplementary file 3 — Description of Additional Supplementary Files [file 41467_2021_22284_MOESM3_ESM.pdf]

Description of Additional Supplementary Files

File Name: Supplementary Data 1
Description: Oligonucleotides for the experiments on DNA origami
