# Supplementary material for: Cellular macromolecules-tethered DNA walking indexing to explore nanoenvironments of chromatin modifications
Source: Nat Commun. 2021 Mar 30;12:1965. doi: 10.1038/s41467-021-22284-z (PMC8009891; doi:10.1038/s41467-021-22284-z)
Supplement: Supplementary file 5 — Reporting Summary [file 41467_2021_22284_MOESM5_ESM.pdf]

nature research

Corresponding author(s): Yongxi Zhao
Last updated by author(s): Feb 22, 2021

Reporting Summary

Nature Research wishes to improve the reproducibility of the work that we publish. This form provides structure for consistency and transparency in reporting. For further information on Nature Research policies, see our Editorial Policies and the Editorial Policy Checklist.

Statistics

For all statistical analyses, confirm that the following items are present in the figure legend, table legend, main text, or Methods section.

n/a	Confirmed	
☐	☒	The exact sample size (*n*) for each experimental group/condition, given as a discrete number and unit of measurement
☐	☒	A statement on whether measurements were taken from distinct samples or whether the same sample was measured repeatedly
☒	☐	The statistical test(s) used AND whether they are one- or two-sided<br>*Only common tests should be described solely by name; describe more complex techniques in the Methods section.*
☒	☐	A description of all covariates tested
☒	☐	A description of any assumptions or corrections, such as tests of normality and adjustment for multiple comparisons
☐	☒	A full description of the statistical parameters including central tendency (e.g. means) or other basic estimates (e.g. regression coefficient) AND variation (e.g. standard deviation) or associated estimates of uncertainty (e.g. confidence intervals)
☒	☐	For null hypothesis testing, the test statistic (e.g. *F*, *t*, *r*) with confidence intervals, effect sizes, degrees of freedom and *P* value noted<br>*Give P values as exact values whenever suitable.*
☒	☐	For Bayesian analysis, information on the choice of priors and Markov chain Monte Carlo settings
☒	☐	For hierarchical and complex designs, identification of the appropriate level for tests and full reporting of outcomes
☒	☐	Estimates of effect sizes (e.g. Cohen's *d*, Pearson's *r*), indicating how they were calculated

Our web collection on statistics for biologists contains articles on many of the points above.

Software and code

Policy information about availability of computer code

Data collection	Fluorescence imaging was performed on a laser scanning confocal microscopy (Leica) with a 63× water objective (NA 1.2). Fluorescence images were acquired as z-stacks at intervals of 0.5 µm. The z-stacks of images were combined to a single one by maximum intensity projection using LAS X Version: 3.1.5.16308. Cells were randomly selected.
Data analysis	Matlab 2019b was used to extracted the spot fluorescence intensity, spot count and the distance of spot to the nucleus periphery of individual cells.

For manuscripts utilizing custom algorithms or software that are central to the research but not yet described in published literature, software must be made available to editors and reviewers. We strongly encourage code deposition in a community repository (e.g. GitHub). See the Nature Research guidelines for submitting code & software for further information.

Data

Policy information about availability of data

All manuscripts must include a data availability statement. This statement should provide the following information, where applicable:

- Accession codes, unique identifiers, or web links for publicly available datasets
- A list of figures that have associated raw data
- A description of any restrictions on data availability

The data that support the findings of this study are available from the corresponding authors on reasonable request. Source data underlying Figs. 2d, 4b, 4c, 5c and 6b are available as a Source data file provided with this paper.

Field-specific reporting

Please select the one below that is the best fit for your research. If you are not sure, read the appropriate sections before making your selection.

- [x] Life sciences
- [ ] Behavioural & social sciences
- [ ] Ecological, evolutionary & environmental sciences

For a reference copy of the document with all sections, see nature.com/documents/nr-reporting-summary-flat.pdf

Life sciences study design

All studies must disclose on these points even when the disclosure is negative.

Sample size	Sample sizes (n= 17; clinical tissues (No. 1-14), FNA samples (No. 15-17)) depended on the availability of patient samples.
Data exclusions	No data were excluded from the analyses.
Replication	All measurements were performed by three or five independent replicates. All attempts at replication were successful.
Randomization	Randomization was not applicable, as all samples were treated equally. Samples were not organized into different experimental groups based on covariates.
Blinding	Investigators were blinded to the experimental group and for data analysis.

Reporting for specific materials, systems and methods

We require information from authors about some types of materials, experimental systems and methods used in many studies. Here, indicate whether each material, system or method listed is relevant to your study. If you are not sure if a list item applies to your research, read the appropriate section before selecting a response.

Materials & experimental systems

n/a	Involved in the study	
[ ]	[x]	Antibodies
[ ]	[x]	Eukaryotic cell lines
[x]	[ ]	Palaeontology and archaeology
[x]	[ ]	Animals and other organisms
[ ]	[x]	Human research participants
[x]	[ ]	Clinical data
[x]	[ ]	Dual use research of concern

Methods

n/a	Involved in the study	
[x]	[ ]	ChIP-seq
[x]	[ ]	Flow cytometry
[x]	[ ]	MRI-based neuroimaging

Antibodies

Antibodies used

Antibodies used in this study:
H3K4me1 (#5326T, Cell Signaling Technology, Danvers, MA, USA), H3K4me3 (#9751T, Cell Signaling Technology, Danvers, MA, USA), H3K27me3 (#9733T, Cell Signaling Technology, Danvers, MA, USA), γH2AX (#9718T, Cell Signaling Technology, Danvers, MA, USA), H3K27ac (#8173T, Cell Signaling Technology, Danvers, MA, USA).Donkey anti-Rabbit IgG (H+L) Highly Cross-Adsorbed Secondary Antibody (#A16037, Invitrogen, Carlsbad, CA) .

Validation

Primary antibodies are used according to the commercial suppliers protocols (Cell Signaling Technology, Danvers, MA, USA):
H3K4me1
Application: W-Western IP-Immunoprecipitation IHC-Immunohistochemistry ChIP-Chromatin Immunoprecipitation IF-Immunofluorescence F-Flow Cytometry E-P-ELISA-PeptideSpecies: H-Human M-Mouse R-Rat Hm-Hamster Mk-Monkey Vir-Virus Mi-Mink C-Chicken Dm-D. melanogaster X-Xenopus Z-Zebrafish B-Bovine Dg-Dog Pg-Pig Sc-S. cerevisiae Ce-C. elegans Hr-Horse All-All Species Expected

H3K4me3
Application: W-Western IP-Immunoprecipitation IHC-Immunohistochemistry ChIP-Chromatin Immunoprecipitation IF-Immunofluorescence F-Flow Cytometry E-P-ELISA-Peptide
Species: H-Human M-Mouse R-Rat Hm-Hamster Mk-Monkey Vir-Virus Mi-Mink C-Chicken Dm-D. melanogaster X-Xenopus Z-Zebrafish B-Bovine Dg-Dog Pg-Pig Sc-S. cerevisiae Ce-C. elegans Hr-Horse All-All Species Expected

H3K27me3
Application: W-Western IP-Immunoprecipitation IHC-Immunohistochemistry ChIP-Chromatin Immunoprecipitation IF-Immunofluorescence F-Flow Cytometry E-P-ELISA-Peptide
Species: H-Human M-Mouse R-Rat Hm-Hamster Mk-Monkey Vir-Virus Mi-Mink C-Chicken Dm-D. melanogaster X-Xenopus Z-Zebrafish

B-Bovine Dg-Dog Pg-Pig Sc-S. cerevisiae Ce-C. elegans Hr-Horse All-All Species Expected

γH2AX
Application: W-Western IP-Immunoprecipitation IHC-Immunohistochemistry ChIP-Chromatin Immunoprecipitation IF-Immunofluorescence F-Flow Cytometry E-P-ELISA-Peptide
Species: H-Human M-Mouse R-Rat Hm-Hamster Mk-Monkey Vir-Virus Mi-Mink C-Chicken Dm-D. melanogaster X-Xenopus Z-Zebrafish B-Bovine Dg-Dog Pg-Pig Sc-S. cerevisiae Ce-C. elegans Hr-Horse All-All Species Expected

H3K27ac
Application: W-Western IP-Immunoprecipitation IHC-Immunohistochemistry ChIP-Chromatin Immunoprecipitation IF-Immunofluorescence F-Flow Cytometry E-P-ELISA-Peptide
Species: H-Human M-Mouse R-Rat Hm-Hamster Mk-Monkey Vir-Virus Mi-Mink C-Chicken Dm-D. melanogaster X-Xenopus Z-Zebrafish B-Bovine Dg-Dog Pg-Pig Sc-S. cerevisiae Ce-C. elegans Hr-Horse All-All Species ExpectedDonkey

anti-Rabbit IgG (H+L) Highly Cross-Adsorbed Secondary Antibody is used according to the commercial suppliers protocols(Invitrogen, Carlsbad, CA) :
Species Reactivity: RabbitHost/Isotype: Donkey / IgGClass: Polyclonal
Applications: ELISA (ELISA) Miscellaneous PubMed (Misc)

Eukaryotic cell lines

Policy information about cell lines

Cell line source(s)	MCF-10A, MCF-7 and MDA-MB-231 cells were all purchased from the ATCC (Manassas, VA, USA).
Authentication	All cell lines were authenticated by the manufacturer (ATCC): ATCC uses morphology, karyotyping, and PCR based approaches to confirm the identity of human cell lines and to rule out both intra- and interspecies contamination. These include an assay to detect species specific variants of the cytochrome C oxidase I gene (COI analysis) to rule out inter-species contamination, and short tandem repeat (STR) profiling to distinguish between individual human cell lines and rule out intraspecies contamination.
Mycoplasma contamination	These cell lines were tested negative for mycoplasma contamination.
Commonly misidentified lines (See ICLAC register)	No commonly misidentified cell lines were used in the study.

Human research participants

Policy information about studies involving human research participants

Population characteristics	The human samples of No. 3, 5-7, 9-11, and 14 are females. Others ate males. The ages of No.1-17 samples are 67, 55, 37, 70, 53, 42, 65, 71, 54, 31, 65, 60, 62, 58, 53, 50, and 65, respectively.
Recruitment	Tissue samples were obtained from Tangdu Hospital of Air Force Medical University. FNA samples were obtained from the Second Affiliated Hospital of Medical College of Xi'an Jiaotong University. Samples were not organized into groups based on gender, age or other covariates. No self-selection bias for patient populations was anticipated.
Ethics oversight	The study of human tissue samples and FNA samples was approved by ethics committee of Xi'an Jiaotong University (No. 2020-216) in accordance with the law on human experiments. Informed consent was obtained from all patients. These clinical tissue and FNA samples were treated with collagenase to collect cell suspensions, and then these cell suspensions were analyzed in the microfluidic chip.

Note that full information on the approval of the study protocol must also be provided in the manuscript.
