# Supplementary material for: Cellular macromolecules-tethered DNA walking indexing to explore nanoenvironments of chromatin modifications
Source: Nat Commun. 2021 Mar 30;12:1965. doi: 10.1038/s41467-021-22284-z (PMC8009891; doi:10.1038/s41467-021-22284-z)
Supplement: Supplementary file 4 — Supplementary Data 1 [file 41467_2021_22284_MOESM4_ESM.zip › Supplementary Data 1.doc]

Oligonucleotides for the experiments on DNA origami

|  | **Oligonucleotides for the experiments on DNA origami** |
| --- | --- |
| 1 | CAAGCCCAATAGGAACCCATGTACAAACAGTT |
| 2 | AATGCCCCGTAACAGTGCCCGTATCTCCCTCA |
| 3 | TGCCTTGACTGCCTATTTCGGAACAGGGATAG |
| 4 | GAGCCGCCCCACCACCGGAACCGCGACGGAAA |
| 5 | AACCAGAGACCCTCAGAACCGCCAGGGGTCAG |
| 6 | TTATTCATAGGGAAGGTAAATATTCATTCAGT |
| 7 | CATAACCCGAGGCATAGTAAGAGCTTTTTAAG |
| 7-overhang | CATAGTGTCCGAAGTCCCGGGGATTTCATAACCCGAGGCATAGTAAGAGCTTTTTAAG |
| 8 | ATTGAGGGTAAAGGTGAATTATCAATCACCGG |
| 9 | AAAAGTAATATCTTACCGAAGCCCTTCCAGAG |
| 10 | GCAATAGCGCAGATAGCCGAACAATTCAACCG |
| 11 | CCTAATTTACGCTAACGAGCGTCTAATCAATA |
| 12 | TCTTACCAGCCAGTTACAAAATAAATGAAATA |
| 13 | ATCGGCTGCGAGCATGTAGAAACCTATCATAT |
| 14 | CTAATTTATCTTTCCTTATCATTCATCCTGAA |
| 15 | GCGTTATAGAAAAAGCCTGTTTAGAAGGCCGG |
| 16 | GCTCATTTTCGCATTAAATTTTTGAGCTTAGA |
| 17 | AATTACTACAAATTCTTACCAGTAATCCCATC |
| 18 | TTAAGACGTTGAAAACATAGCGATAACAGTAC |
| 19 | TAGAATCCCTGAGAAGAGTCAATAGGAATCAT |
| 20 | CTTTTACACAGATGAATATACAGTAAACAATT |
| 21 | TTTAACGTTCGGGAGAAACAATAATTTTCCCT |
| 22 | CGACAACTAAGTATTAGACTTTACAATACCGA |
| 23 | GGATTTAGCGTATTAAATCCTTTGTTTTCAGG |
| 24 | ACGAACCAAAACATCGCCATTAAATGGTGGTT |
| 25 | GAACGTGGCGAGAAAGGAAGGGAACAAACTAT |
| 26 | TAGCCCTACCAGCAGAAGATAAAAACATTTGA |
| 27 | CGGCCTTGCTGGTAATATCCAGAACGAACTGA |
| 28 | CTCAGAGCCACCACCCTCATTTTCCTATTATT |
| 29 | CTGAAACAGGTAATAAGTTTTAACCCCTCAGA |
| 30 | AGTGTACTTGAAAGTATTAAGAGGCCGCCACC |
| 31 | GCCACCACTCTTTTCATAATCAAACCGTCACC |
| 32 | GTTTGCCACCTCAGAGCCGCCACCGATACAGG |
| 33 | GACTTGAGAGACAAAAGGGCGACAAGTTACCA |
| 34 | AGCGCCAACCATTTGG GAATTAGATTATTAGC |
| 35 | GAAGGAAAATAAGAGC AAGAAACAACAGCCAT |
| 36 | GCCCAATACCGAGGAAACGCAATAGGTTTACC |
| 37 | ATTATTTAACCCAGCTACAATTTTCAAGAACG |
| 38 | TATTTTGCTCCCAATCCAAATAAGTGAGTTAA |
| 39 | GGTATTAAGAACAAGAAAAATAATTAAAGCCA |
| 40 | TAAGTCCTACCAAGTACCGCACTCTTAGTTGC |
| 41 | ACGCTCAAAATAAGAATAAACACCGTGAATTT |
| 42 | AGGCGTTACAGTAGGGCTTAATTGACAATAGA |
| 43 | ATCAAAATCGTCGCTATTAATTAACGGATTCG |
| 44 | CTGTAAATCATAGGTCTGAGAGACGATAAATA |
| 45 | CCTGATTGAAAGAAATTGCGTAGACCCGAACG |
| 46 | ACAGAAATCTTTGAATACCAAGTTCCTTGCTT |
| 47 | TTATTAATGCCGTCAATAGATAATCAGAGGTG |
| 48 | AGATTAGATTTAAAAGTTTGAGTACACGTAAA |
| 49 | AGGCGGTCATTAGTCTTTAATGCGCAATATTA |
| 50 | GAATGGCTAGTATTAACACCGCCTCAACTAAT |
| 51 | CCGCCAGCCATTGCAACAGGAAAAATATTTTT |
| 52 | CCCTCAGAACCGCCACCCTCAGAACTGAGACT |
| 53 | CCTCAAGAATACATGGCTTTTGATAGAACCAC |
| 54 | TAAGCGTCGAAGGATTAGGATTAGTACCGCCA |
| 55 | CACCAGAGTTCGGTCATAGCCCCCGCCAGCAA |
| 56 | TCGGCATTCCGCCGCCAGCATTGACGTTCCAG |
| 57 | AATCACCAAATAGAAAATTCATATATAACGGA |
| 58 | TCACAATCGTAGCACCATTACCATCGTTTTCA |
| 59 | ATACCCAAGATAACCCACAAGAATAAACGATT |
| 60 | ATCAGAGAAAGAACTGGCATGATTTTATTTTG |
| 61 | TTTTGTTTAAGCCTTAAATCAAGAATCGAGAA |
| 62 | AGGTTTTGAACGTCAAAAATGAAAGCGCTAAT |
| 63 | CAAGCAAGACGCGCCTGTTTATCAAGAATCGC |
| 64 | AATGCAGACCGTTTTTATTTTCATCTTGCGGG |
| 65 | CATATTTAGAAATACCGACCGTGTTACCTTTT |
| 66 | AATGGTTTACAACGCCAACATGTAGTTCAGCT |
| 67 | TAACCTCCATATGTGAGTGAATAAACAAAATC |
| 68 | AAATCAATGGCTTAGGTTGGGTTACTAAATTT |
| 69 | GCGCAGAGATATCAAAATTATTTGACATTATC |
| 70 | AACCTACCGCGAATTATTCATTTCCAGTACAT |
| 71 | ATTTTGCGTCTTTAGGAGCACTAAGCAACAGT |
| 72 | CTAAAATAGAACAAAGAAACCACCAGGGTTAG |
| 73 | GCCACGCTATACGTGGCACAGACAACGCTCAT |
| 74 | GCGTAAGAGAGAGCCAGCAGCAAAAAGGTTAT |
| 75 | GGAAATACCTACATTTTGACGCTCACCTGAAA |
| 76 | GATAAGTGAATAGGTGTATCACCGTACTCAGGAGGTTTAGCGGGGTTT |
| 77 | TGCTCAGTCAGTCTCTGAATTTACCAGGAGGT |
| 79 | TGAGGCAGGCGTCAGACTGTAGCGTAGCAAGG |
| BioT80 | ATTGGCCTGCCAGAATGGAAAGCGACCAGGCGTTATTATTA  TTATTATTATTA |
| 81 | CCGGAAACACACCACGGAATAAGTAAGACTCC |
| 82 | ACGCAAAGGTCACCAATGAAACCAATCAAGTT TGCCTTTAGTCAGACG |
| 83 | TTATTACGGTCAGAGGGTAATTGAATAGCAGC |
| 84 | TGAACAAACAGTATGTTAGCAAACTAAAAGAA |
| 85 | CTTTACAGTTAGCGAACCTCCCGACGTAGGAA |
| 86 | GAGGCGTTAGAGAATAACATAAAAGAACACCC |
| 87 | TCATTACCCGACAATAAACAACATATTTAGGC |
| 88 | CCAGACGAGCGCCCAATAGCAAGCAAGAACGC |
| 89 | AGAGGCATAATTTCATCTTCTGACTATAACTA |
| 90 | TTTTAGTTTTTCGAGCCAGTAATAAATTCTGT |
| 91 | TATGTAAACCTTTTTTAATGGAAAAATTACCT |
| 92 | TGAAACAAAATTTCAT TTGAATTATGCTGATG CAAATCCACAAATATA |
| 93 | GAGCAAAAACTTCTGAATAATGGAAGAAGGAG |
| 95 | CGGAATTATTGAAAGGAATTGAGGTGAAAAAT |
| BioT96 | TTCCTGATTGATTGTTTGGATTATGAAGATGATTATTATTATTATTATTATTA |
| 97 | CTAAAGCAAGATAGAACCCTTCTGAATCGTCT |
| 98 | GCCAACAGTCACCTTG CTGAACCTGTTGGCAA ATCAACAGTCATCATA |
| 99 | GAAATGGATTATTTACATTGGCAGACATTCTG |
| 100 | TTTTTATAAGTATAGCCCGGCCGTCGAG |
| 101 | AGGGTTGATTTTATAAATCCTCATTAAATGATATTC |
| 102 | ACAAACAATTTTAATCAGTAGCGACAGATCGATAGC |
| 103 | AGCACCGTTTTTTAAAGGTGGCAACATAGTAGAAAA |
| 104 | TACATACATTTTGACGGGAGAATTAACTACAGGGAA |
| 105 | GCGCATTATTTTGCTTATCCGGTATTCTAAATCAGA |
| 106 | TATAGAAGTTTTCGACAAAAGGTAAAGTAGAGAATA |
| 107 | TAAAGTACTTTTCGCGAGAAAACTTTTTATCGCAAG |
| 108 | ACAAAGAATTTTATTAATTACATTTAACACATCAAG |
| 109 | AAAACAAATTTTTTCATCAATATAATCCTATCAGAT |
| 110 | GATGGCAATTTTAATCAATATCTGGTCACAAATATC |
| 111 | AAACCCTCTTTTACCAGTAATAAAAGGGATTCACCAGTCACACGTTTT |
| 112 | CCGAAATCCGAAAATCCTGTTTGAAGCCGGAA |
| 113 | CCAGCAGGGGCAAAATCCCTTATAAAGCCGGC |
| 114 | GCATAAAGTTCCACACAACATACGAAGCGCCA |
| 115 | GCTCACAATGTAAAGCCTGGGGTGGGTTTGCC |
| 116 | TTCGCCATTGCCGGAAACCAGGCATTAAATCA |
| 117 | GCTTCTGGTCAGGCTGCGCAACTGTGTTATCC |
| 118 | GTTAAAATTTTAACCAATAGGAACCCGGCACC |
| 118-Near | GAGGACAACGATTATAACAGTTTTTTGTTAAAATTTTAACCAATAGGAACCCGGCACC |
| 119 | AGACAGTCATTCAAAAGGGTGAGAAGCTATAT |
| 120-overhang | AGGTAAAGAAATCACCATCAATATAATATTTT |
| 120 | CATAGTGTCCGAAGTCCCGGGGATTTAGGTAAAGAAATCACCATCAATATAATATTTT |
| 121 | TTTCATTTGGTCAATAACCTGTTTATATCGCG |
| 122 | CACTCACGTAACTCATGTCAAGGTTTTCGCAAATGGGGCGCGAGCTGAAATAATGTGT |
| 123 | TTTTAATTGCCCGAAAGACTTCAAAACACTAT |
| 123-overhang | TTTTTCGCATAGTAGTAGTTAGGTTTTTTTAATTGCCCGAAAGACTTCAAAACACTAT |
| 124 | CTGTCTACACTCTGACTCTGACTTTTAAGAGGAACGAGCTTCAAAGCGAAGATACATT |
| 125 | GGAATTACTCGTTTACCAGACGACAAAAGATT |
| 125-overhang | TTTTTCGTCTGACCATGGATGCCTTTGGAATTACTCGTTTACCAGACGACAAAAGATT |
| 126 | GAATAAGGACGTAACAAAGCTGCTCTAAAACA |
| 127 | CCAAATCACTTGCCCTGACGAGAACGCCAAAA |
| 128 | CTCATCTTGAGGCAAAAGAATACAGTGAATTT |
| 129 | AAACGAAATGACCCCCAGCGATTATTCATTAC |
| 130 | CTTAAACATCAGCTTGCTTTCGAGCGTAACAC |
| 131 | TCGGTTTAGCTTGATACCGATAGTCCAACCTA |
| 132 | TGAGTTTCGTCACCAGTACAAACTTAATTGTA |
| 133 | CCCCGATTTAGAGCTTGACGGGGAAATCAAAA |
| 134 | GAATAGCCGCAAGCGGTCCACGCTCCTAATGA |
| 135 | GAGTTGCACGAGATAGGGTTGAGTAAGGGAGC |
| 136 | GTGAGCTAGTTTCCTGTGTGAAATTTGGGAAG |
| 137 | TCATAGCTACTCACATTAATTGCGCCCTGAGA |
| 138 | GGCGATCGCACTCCAGCCAGCTTTGCCATCAA |
| 139 | GAAGATCGGTGCGGGCCTCTTCGCAATCATGG |
| 140 | AAATAATTTTAAATTGTAAACGTTGATATTCA |
| 141 | GCAAATATCGCGTCTGGCCTTCCTGGCCTCAG |
| 142 | ACCGTTCTAAATGCAATGCCTGAGAGGTGGCA |
| 143 | TATATTTTAGCTGATAAATTAATGTTGTATAA |
| 144 | TCAATTCTTTTAGTTTGACCATTACCAGACCG |
| 145 | CGAGTAGAACTAATAGTAGTAGCAAACCCTCA |
| 146 | GAAGCAAAAAAGCGGATTGCATCAGATAAAAA |
| 146-overhang | GAGGACAACGATTATAACAGTTTTTTGAAGCAAAAAAGCGGATTGCATCAGATAAAAA |
| 147 | TCAGAAGCCTCCAACAGGTCAGGATCTGCGAA |
| 148 | CCAAAATATAATGCAGATACATAAACACCAGA |
| 148-overhang | TTTTTAGTTCGACGTACTGCACATTTCCAAAATATAATGCAGATACATAAACACCAGA |
| 149 | CATTCAACGCGAGAGGCTTTTGCATATTATAG |
| 150 | ACGAGTAGTGACAAGAACCGGATATACCAAGC |
| 151 | AGTAATCTTAAATTGGGCTTGAGAGAATACCA |
| 152 | GCGAAACATGCCACTACGAAGGCATGCGCCGA |
| 153 | ATACGTAAAAGTACAACGGAGATTTCATCAAG |
| 154 | CAATGACACTCCAAAAGGAGCCTTACAACGCC |
| 155 | AAAAAAGGACAACCATCGCCCACGCGGGTAAA |
| 156 | TGTAGCATTCCACAGACAGCCCTCATCTCCAA |
| 157 | GTAAAGCACTAAATCGGAACCCTAGTTGTTCC |
| 158 | AGTTTGGAGCCCTTCACCGCCTGGTTGCGCTC |
| 159 | AGCTGATTACAAGAGTCCACTATTGAGGTGCC |
| 160 | ACTGCCCGCCGAGCTCGAATTCGTTATTACGC |
| 161 | CCCGGGTACTTTCCAGTCGGGAAACGGGCAAC |
| 162 | CAGCTGGCGGACGACGACAGTATCGTAGCCAG |
| 163 | GTTTGAGGGAAAGGGGGATGTGCTAGAGGATC |
| 164 | CTTTCATCCCCAAAAACAGGAAGACCGGAGAG |
| 165 | AGAAAAGCAACATTAAATGTGAGCATCTGCCA |
| 166 | GGTAGCTAGGATAAAAATTTTTAGTTAACATC |
| 167 | CAACGCAATTTTTGAGAGATCTACTGATAATC |
| 168 | CAATAAATACAGTTGATTCCCAATTTAGAGAG |
| 169 | TCCATATACATACAGGCAAGGCAACTTTATTT |
| 170 | TACCTTTAAGGTCTTTACCCTGACAAAGAAGT |
| 171 | CAAAAATCATTGCTCCTTTTGATAAGTTTCAT |
| 172 | TTTGCCAGATCAGTTGAGATTTAGTGGTTTAA |
| 173 | AAAGATTCAGGGGGTAATAGTAAACCATAAAT |
| 174 | TTTCAACTATAGGCTGGCTGACCTTGTATCAT |
| 175 | CCAGGCGCTTAATCATTGTGAATTACAGGTAG |
| 176 | CGCCTGATGGAAGTTTCCATTAAACATAACCG |
| 177 | TTTCATGAAAATTGTGTCGAAATCTGTACAGA |
| 178 | ATATATTCTTTTTTCACGTTGAAAATAGTTAG |
| 179 | AATAATAAGGTCGCTGAGGCTTGCAAAGACTT |
| 180 | CGTAACGATCTAAAGTTTTGTCGTGAATTGCG |
| 181 | ACCCAAATCAAGTTTTTTGGGGTCAAAGAACG |
| 182 | TGGACTCCCTTTTCACCAGTGAGACCTGTCGT |
| 183 | AATCGGCCCGCCAGGGTGGTTTTTAACGTCAA AGGGCGAAGAACCATC |
| 184 | GCCAGCTGCCTGCAGGTCGACTCTGCAAGGCG |
| 186 | ATTAAGTTCGCATCGTAACCGTGCGAGTAACA |
| BioT187 | CCAGGGTTGTGCCAAGCTTGCATGCATTAATGTTATTATTATTATTATTATTA |
| 188 | ACCCGTCGTCATATGTACCCCGGTAAAGGCTA |
| 189 | CATGTCAAGATTCTCCGTGGGAACCGTTGGTG TAGATGGGGGGTAACG |
| 190 | TCAGGTCACTTTTGCGGGAGAAGCAGAATTAG |
| 191 | CTGTAATATTGCCTGAGAGTCTGGAAAACTAG |
| 192 | CAAAATTAAAGTACGGTGTCTGGAAGAGGTCA |
| 193 | TGCAACTAAGCAATAAAGCCTCAGTTATGACC |
| 194 | TTTTTGCGCAGAAAACGAGAATGAATGTTTAG |
| 195 | AAACAGTTGATGGCTTAGAGCTTATTTAAATA |
| 196 | ACTGGATAACGGAACAACATTATTACCTTATG |
| 197 | CTCATTATTTAATAAA ACGAACTAGCGTCCAATACTGCGGAATGCTTT |
| 198 | CGATTTTAGAGGACAG ATGAACGGCGCGACCT |
| 200 | GCTCCATGAGAGGCTTTGAGGACTAGGGAGTT |
| BioT201 | CCGGAACGCTGACCAACTTTGAAAAGAACTGGTTATTATTATTATTATTATTA |
| 202 | AAAGGCCGAAAGGAACAACTAAAGCTTTCCAG |
| 203 | GAGAATAGCTTTTGCGGGATCGTCGGGTAGCA  ACGGCTACTTACTTAG |
| 204 | ACGTTAGTAAATGAATTTTCTGTAAGCGGAGT |
| 205 | TTTTCGATGGCCCACTACGTAAACCGTC |
| 206 | TATCAGGGTTTTCGGTTTGCGTATTGGGAACGCGCG |
| 207 | GGGAGAGGTTTTTGTAAAACGACGGCCATTCCCAGT |
| 208 | CACGACGTTTTTGTAATGGGATAGGTCAAAACGGCG |
| 209 | GATTGACCTTTTGATGAACGGTAATCGTAGCAAACA |
| 210 | AGAGAATCTTTTGGTTGTACCAAAAACAAGCATAAA |
| 211 | GCTAAATCTTTTCTGTAGCTCAACATGTATTGCTGA |
| 212 | ATATAATGTTTTCATTGAATCCCCCTCAAATCGTCA |
| 213 | TAAATATTTTTTGGAAGAAAAATCTACGACCAGTCA |
| 214 | GGACGTTGTTTTTCATAAGGGAACCGAAAGGCGCAG |
| 215 | ACGGTCAATTTTGACAGCATCGGAACGAACCCTCAG |
| 216 | CAGCGAAATTTTAACTTTCAACAGTTTCTGGGATTTTGCTAAACTTTT |
| Biotin probe | **Biotin**-TAATAATAATAATAATAATAA |
|  |  |
| Walking probe (spacer 0nt) on origami | AGTCAGAGTCAGAGTGTAGACAGTAAAAAAAAAAAAAAAAAAAAAAAAAAAAAAAAAAAAAAACT***GCTGAGG***CACAT |
| Walking probe (spacer 15nt) on origami | AGTCAGAGTCAGAGTGTAGACAGTAAAAAAAAAAAAAAAAAAAAAAAAAAAAAAAAAAAAAAAAAAAAAAAAAAAAAACT***GCTGAGG***CACAT |
| Walking probe (spacer 30nt) on origami | AGTCAGAGTCAGAGTGTAGACAGTAAAAAAAAAAAAAAAAAAAAAAAAAAAAAAAAAAAAAAAAAAAAAAAAAAAAAAAAAAAAAAAAAAAAACT***GCTGAGG***CACAT |
| Barcoding probe-5hmC on origami | AAACTGTTATAATCGTTGTCCTCGTAAATCCTCATCAATCA*T*CTATGTG***CCTCAGC***AGAGGAGA*A*T*C*A*C*G |
| Barcoding probe-5hmU on origami | CCTTGACATGAGTTACGTGAGTGCAGCAAACGGGAAGAGTC*T*TTATGTG***CCTCAGC***AGAGGAGA*A*T*C*A*C*G |
| Barcoding probe-5fU on origami | TCCCCGGGACTTCGGACACTATGTTTCTTGAGGAGGGCAGC*A*ATATGTG***CCTCAGC***AGAGGAGA*A*T*C*A*C*G |
| Mismatched Barcoding probe-5fU on origami | AAAAAAAAAAAAAAAAAACTATGTTTCTTGAGGAGGGCAGC*A*ATATGTG***CCTCAGC***AGAGGAGA*A*T*C*A*C*G |
|  |  |

The symbol ‘*’ indicates phosphorothioate. Italic letters in red indicate the nick sequence of Nt.BbvCI enzyme.
